# Genetic Variation in the Magnitude and Longevity of the IgG Subclass Response to a Diphtheria-Tetanus-Acellular Pertussis (DTaP) Vaccine in Mice

**DOI:** 10.3390/vaccines7040124

**Published:** 2019-09-20

**Authors:** Yung-Yi C. Mosley, Josiah E. Radder, Harm HogenEsch

**Affiliations:** 1 Department of Comparative Pathobiology, College of Veterinary Medicine, Purdue University, West Lafayette, IN 47907 USA; chen37@purdue.edu; 2 Department of Medicine, Division of Pulmonary, Allergy and Critical Care Medicine, University of Pittsburgh, Pittsburgh, PA 15219, USA; jeradder@gmail.com; 3 Purdue Institute of Inflammation, Immunology, and Infectious Diseases, Purdue University, Indiana Purdue University, West Lafayette, IN 47907, USA

**Keywords:** DTaP, vaccine, IgG subclass, antibody magnitude, antibody longevity, genetics

## Abstract

The type of IgG subclasses induced by vaccination is an important determinant of vaccine efficacy because the IgG subclasses vary in their biological function. The goal of this study was to determine the influence of the genetic background on the production and duration of vaccine-induced IgG subclasses. IgG1, IgG2b, and IgG3 titers against diphtheria toxoid (DT), pertussis toxin (PT), filamentous hemagglutinin (FHA), and pertactin (Prn) were measured in mice from 28 different inbred and wild-derived strains vaccinated with an aluminum hydroxide-adjuvanted DTaP vaccine. The titers and duration of vaccine-specific IgG subclass responses were different among mouse strains, indicating that genetic factors contribute to this variation. Statistical associations were used to identify potential mechanisms that contribute to antibody production and longevity. This analysis showed that the mechanisms guiding the magnitude of antibody production were antigen-dependent for IgG1 but antigen-independent for IgG2b and IgG3. However, the mechanisms driving the longevity of antibody titers were antigen-independent for IgG1, IgG2b, and IgG3. The ratio of IgG1 and IgG3 titers identified Th1 and Th2-prone mouse strains. TLR4-deficient C3H/HeJ mice had an enhanced IgG1 response compared with C3H/HeOuJ mice with intact TLR4. This work demonstrates that the genetic background contributes significantly to the magnitude and longevity of vaccine-induced IgG1, IgG2b, and IgG3 titers in mice.

## 1. Introduction 

Human and mouse immunoglobulins are classified into five isotypes, i.e., IgA, IgD, IgE, IgG, and IgM, which differ in their heavy chain structure and corresponding effector function [1]. In mice, IgG is further divided into IgG1, IgG2a, IgG2b, IgG2c, and IgG3 subclasses. Except for IgG2a and IgG2c, IgG subclasses are shared among inbred mouse strains. Inbred mice either have an *Igh-1a* or *Igh-1b* allele at their heavy chain locus which encodes for IgG2a and IgG2c, respectively [2,3,4]. For example, BALB/cJ and C3H/HeJ mice express IgG2a, whereas C57BL/6J and NOD/ShiLtJ mice have IgG2c [3,5]. The different IgG subclasses exert their biological functions such as antibody-dependent cell-mediated phagocytosis, antibody-dependent cellular cytotoxicity, complement-dependent cell-mediated cytotoxicity and complement-dependent cell-mediated phagocytosis through the binding of IgG Fc receptors (FcγR) and the C1q molecule [1,6]. The different biological functions of the IgG subclasses are related to their affinity for specific FcγRs and C1q [6,7,8,9]. 

During the immune response, B cells undergo isotype switching from IgM and IgD to IgA, IgE, or one of the IgG subclasses. The underlying process, class switch recombination, is linked to cell division and is regulated by activation-induced cytidine deaminase [10]. The decision of which isotype or IgG subclass to switch to is determined by the imprinted state as well as the prevailing cytokine milieu [11]. In mice, IFNγ induces isotype switching to IgG2a/c and IgG3; IL-4 to IgG1 and IgE; and TGF-β to IgG2b and IgA [12,13,14]. As IL-4 is typically associated with Th2 cells and IFNγ with Th1 cells, the relative levels of IgG subclasses can be used as an approximation to determine bias in the immune response towards Th1 or Th2 cells [12,15]. 

Under the same vaccination regimen, profound inter-individual variability is observed in the immune response to vaccines, including the childhood vaccine for pertussis [16,17,18,19]. Twin studies have shown that the inter-individual differences in antibody titers in response to childhood vaccination can be partially attributed to genetic determinants with heritability ranging from 36 to 77% [20,21,22,23]. Understanding the genetic basis of the immune response to vaccination may provide new mechanistic insights and lead to improved vaccines and vaccination strategies. We and others have exploited the classical inbred strain association method in mice to reveal the genetic basis for physiological traits and immune responses [24,25,26]. The employment of the mouse model system in a genome-wide association study has benefits such as well-controlled environmental factors and relatively low cost as genotyping and breeding are not required, but it also suffers from low genome-wide power and potential false-positive associations [27,28,29]. Statistical mixed linear models can reduce false-positive associations due to genetic relatedness [29,30]. In a previous study, we observed marked variation in the titer, avidity and longevity of IgG antibodies against diphtheria toxoid (DT), pertussis toxoid (PT), filamentous hemagglutinin (FHA), and pertactin (Prn) in 28 inbred strains of mice following vaccination with a licensed diphtheria-tetanus-acellular pertussis (DTaP) vaccine [26]. There was a strong correlation between the longevity of the IgG responses specific for the four antigens, whereas the variation in the titer and avidity of the IgG were antigen-specific. Although the exact correlates of protection in pertussis remain to be determined, antibodies are thought to play an important role [31,32]. Antibodies enhanced the phagocytosis of *Bordetella pertussis* in a FcγR-dependent manner in human neutrophils [33]. Therefore, the frequency and duration of IgG subclass responses is likely to be an important determinant of the protection provided by pertussis vaccination. Previous mouse studies have shown that the IgG response to vaccination with DTaP is dominated by IgG1 [34,35]. However, C57BL/6 mice also developed substantial IgG2c titers [34] whereas IgG subclasses other than IgG1 were reported undetectable in BALB/c mice [35]. Although these were separate studies with different experimental protocols, the results are consistent with a bias towards Th1 responses in C57BL/6 mice and towards Th2 in BALB/c mice [36,37], and suggest genetic variation in the production of IgG subclasses in response to DTaP vaccination. Here, we investigated the level and maintenance of IgG subclass titers in serum samples collected from the 28 mouse strains immunized with DTaP [26]. To the best of our knowledge, this is the first time that such a large cohort of genetically diverse inbred strains of mice was immunized with a vaccine in a single study. Utilizing the cohort data, we performed association analysis to reveal characteristics of the IgG subclass response toward the DTaP vaccine. We further used the identified genetic linkages to validate these findings.

## 2. Material and Methods

### 2.1. Mice and Vaccination

The experimental protocol was previously described [26]. Briefly, five-week-old female mice (n = 8) of 28 different inbred strains (Jackson Laboratory, Bar Harbor, ME, USA) were vaccinated three times intramuscularly with 50 µL of a diphtheria-tetanus-acellular pertussis vaccine (DTaP) (Infanrix^®^, GlaxoSmithKline, Rixensart, Belgium) at 6, 8, and 12 weeks of age in alternating hind legs. Blood samples were collected at 14 and 24 weeks of age. The protocol was approved by the Purdue University Animal Care and Use Committee (protocol 1304000853).

### 2.2. Serology by ELISA

Serology was performed essentially as described [26]. Briefly, microtiter plates were coated with 2 µg/mL diphtheria toxoid (DT), tetanus toxoid, pertussis toxin (PT), filamentous hemagglutinin (FHA), or pertactin (Prn) (List Biological, Campbell, CA, USA) in 50 mM carbonate buffer (pH 9.6) at 4°C overnight. Wells were blocked with 1% BSA in PBST, and incubated with diluted serum samples followed by HRP-conjugated secondary antibody against IgG1, IgG2b or IgG3 (Southern Biotech, Birmingham, AL, USA). The reaction was revealed by TMB substrate (Sigma-Aldrich, St. Louis, MO, USA), stopped with 2N H_2_SO_4_ and read at 450 nm (BioTek, Winooski, VT, USA). Serum from BALB/cJ mice hyperimmunized with DTaP was used to develop a standard curve in each plate. The titer of IgG1, IgG2b, and IgG3 in the hyperimmune serum was set at 100,000, 10,000 and 1000 units/mL, respectively. Antibody titers less than the cutoff (≤ 800 U/mL for IgG1; ≤ 200 U/mL for IgG2b and IgG3) were assigned a titer of half of the cutoff. Antibody magnitude measured at week14 was presented after log_10_ transformation and antibody longevity was calculated as log_10_ (titer at week24/titer at week14).

### 2.3. Genetic Association Mapping

We tested for association between magnitude and longevity of IgG1, IgG2b, and IgG3 antibody responses and four million single nucleotide polymorphisms (SNPs) published by the National Institute of Environmental Health Sciences (NIEHS) using the genome-wide efficient mixed model association (GEMMA) [30]. Association testing excluded the C3H/HeOuJ strain as this strain has not been independently genotyped. When published, these SNPs were aligned to NCBI37/mm9, and this is reflected in all genomic locations reported here. Genes with any coding material within one million base pairs (1Mb) of each of the SNPs with suggestive association (*p* < 0.001) for each phenotype were identified. Venn diagrams (Venny 2.1, http://bioinfogp.cnb.csic.es/tools/venny/index.html) were applied to demonstrate the number of common genes shared by the four vaccine antigens DT, PT, FHA and Prn.

### 2.4. Statistical Analysis

Data are presented as mean ± SEM. The statistical significance of differences between groups was determined by Student’s t test or one-way ANOVA followed by Tukey’s multiple comparison test using GraphPad Prism 7 (GraphPad software, La Jolla, CA, USA). Since DBA/1J mice were not individually identified, the longevity analysis was omitted for this strain. The IgG1 to IgG3 ratio was calculated as log_10_ (IgG1 titer at week 14/IgG3 titer at week 14). The longevity of IgG1, IgG2b, and IgG3 was calculated by the ratio between titers at wk14 and wk24 as log_10_ (wk24/wk14). Data points less than the cutoff value were removed from longevity calculation. For correlation analysis, Pearson’s correlation coefficient (r) was calculated from the mean value of each strain and presented with the *p*-value.

## 3. Results

### 3.1. Significant Inter-Strain Variability for IgG1, IgG2b, and IgG3

**Figure 1:**
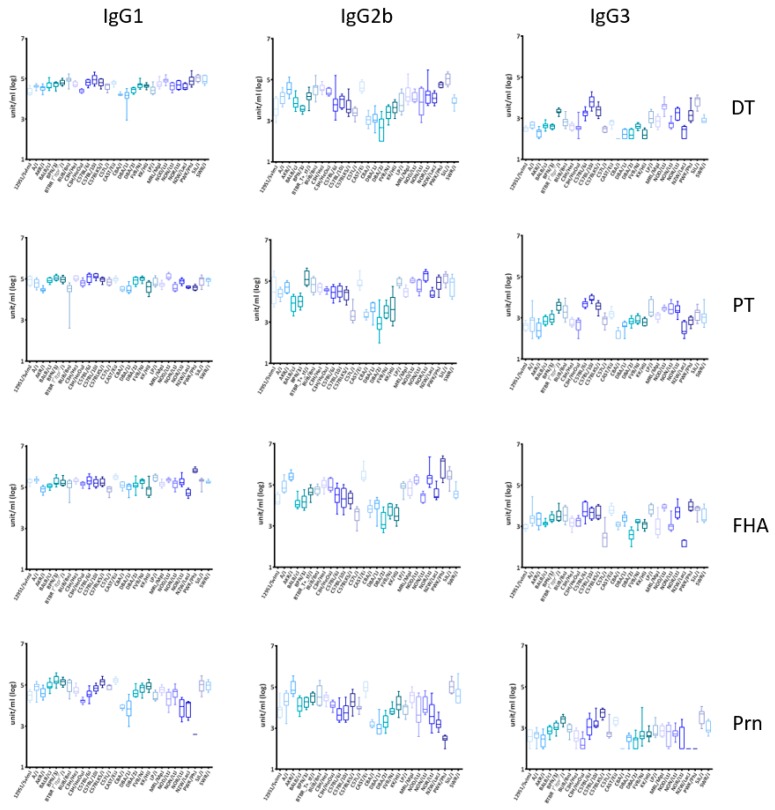
IgG1, IgG2b, and IgG3 titers to Diphtheria-Tetanus-Acellular Pertussis (DTaP) vaccine antigens in inbred mouse strains. Titers were determined at week 14 (two weeks after the third vaccination) against four antigens in the DTaP vaccine by ELISA. There was significant variation (*p* < 0.0001 by ANOVA) between the antibody magnitude from 28 strains of mice for diphtheria toxoid (DT), pertussis toxin (PT), filamentous hemagglutinin (FHA), and pertactin (Prn) for each IgG subclass.

The DTaP vaccine used in this study contains five antigens formulated with aluminum hydroxide adjuvant. The IgG1, IgG2b, and IgG3 titers to DT and the pertussis antigens PT, FHA and Prn were measured in serum samples collected at week 14 and 24, two and 12 weeks after the third vaccination, respectively. We did not determine IgG2a and IgG2c subclasses because of their variable expression among mouse strains. The mice generally had high titers of IgG1, moderate titers of IgG2b and low titers of IgG3 at week 14 (Figure 1). Longevity of the IgG was determined as the log10 of the ratio of the titers at week 24 divided by the titer at week 14 (Figure S1). The antibody titers at week 24 are presented in Figure S2. For all three IgG subclasses, the titers and longevity of the antigen-specific responses varied significantly among mouse strains (*p* < 0.0001), indicating that genetic factors contribute to variations in the antibody phenotypes across mouse strains. 

### 3.2. Contrasting Patterns of Antibody Titers and Longevity Correlations Between IgG1, IgG2b, and IgG3

**Figure 2:**
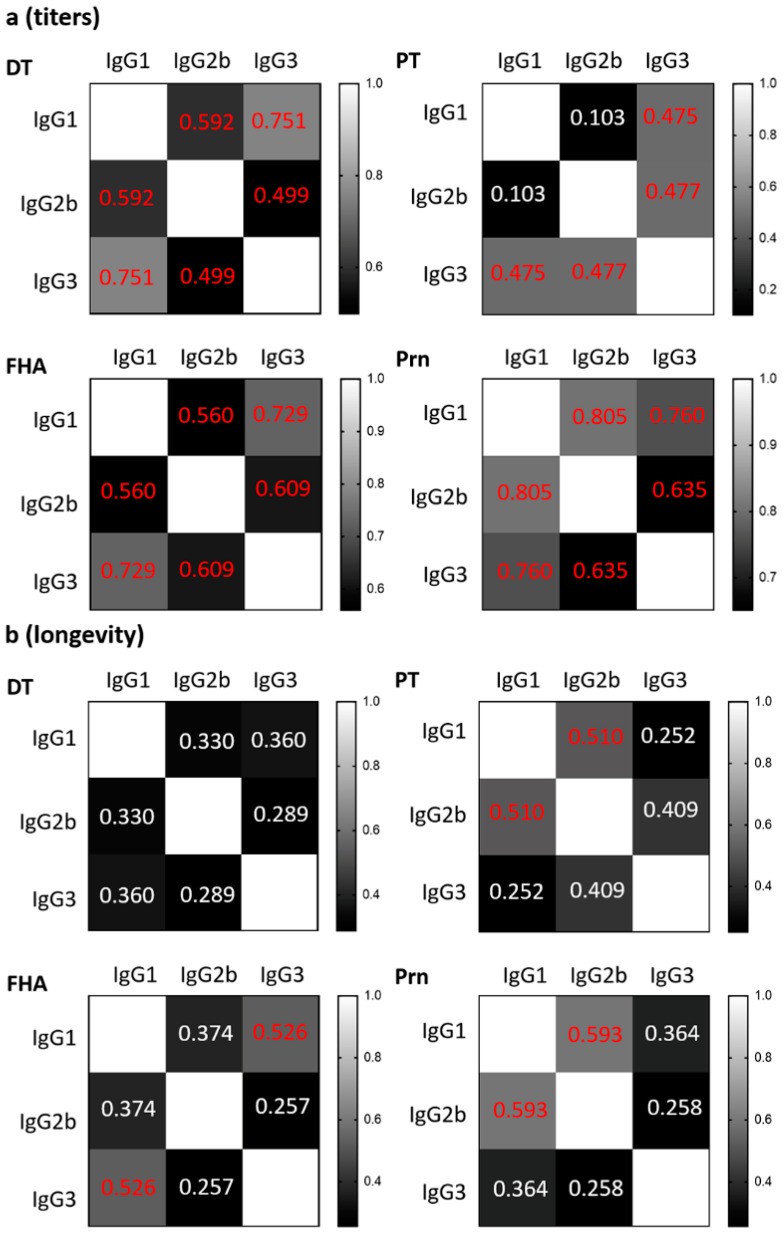
Correlations between IgG1, IgG2b, and IgG3 against the same vaccine antigen. The correlations between IgG1, IgG2b, and IgG3 were plotted against the same vaccine antigen for antibody magnitude (**a**) and longevity (**b**). Mean value of the longevity of each strain was used to calculate for Pearson’s correlation coefficient (r) for the correlations between the magnitude and longevity of IgG1 and IgG2b, IgG1 and IgG3, IgG2b and IgG3. Significant values (*p* < 0.05) are indicated in red font.

The correlations between IgG1, IgG2b, and IgG3 were plotted for each vaccine antigen for antibody titers (Figure S3) and longevity (Figure S4) and the data are summarized in Figure 2. There were significant medium to strong correlations between IgG subclass titers to the same antigen, except for PT-specific IgG1 and IgG2b. This indicates that the level of the antibody response to a specific vaccine antigen is reflected across IgG subclasses, i.e., a higher IgG1 titer correlates with a higher IgG2b and IgG3 titer to the same antigen. These positive correlations suggest that the level of antibody production is largely determined by the immunogenicity of the antigen regardless of which IgG subclass was induced. In contrast, the longevity of IgG subclasses to the same vaccine antigen did not correlate with each other, or only with a low to medium level of significance (Figure 2b and Figure S4), e.g., a longer duration of PT-specific IgG1 did not point toward a longer duration of IgG2b nor IgG3 to PT. The poor correlation between the IgG subclasses for longevity suggests that the mechanisms regulating the duration of antibody titers are different from antibody magnitude and that antibody longevity is regulated by factors other than the immunogenicity of the vaccine antigens.

### 3.3. Different Patterns of Antigen-Specific Responses Among IgG1, IgG2b, and IgG3 for Magnitude and Longevity

**Figure 3:**
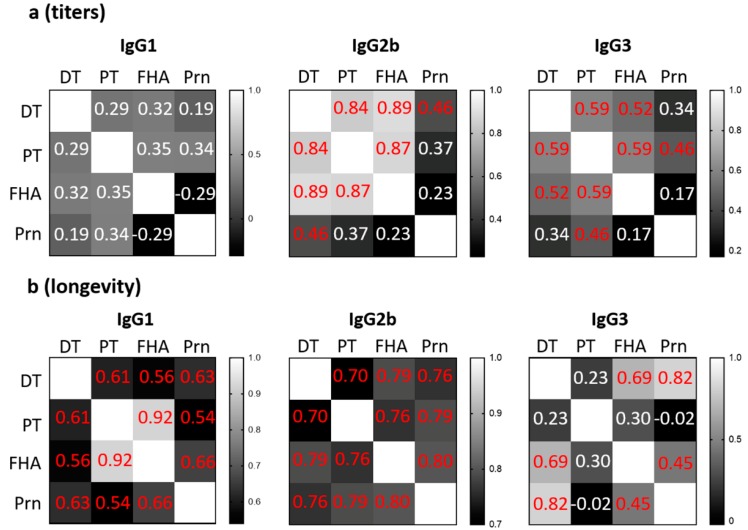
Correlations between antigen-specific responses among IgG1, IgG2b, and IgG3. The correlations between antibody response to different vaccine antigens for IgG1, IgG2b, and IgG3 were plotted for antibody magnitude (**a**) and longevity (**b**). The correlations were presented by Pearson r values for each pair of antigen-specific antibody within the same IgG subclass. Significant values (*p* < 0.05) are indicated in red font.

Since DTaP is a multivalent vaccine, we sought to further dissect the antibody response to different antigens in the vaccine. Pearson correlations between antibody responses against different vaccine antigens were calculated for the titer (Figure 3a and Figure S5) and longevity (Figure 3b and Figure S6). There was no significant correlation for IgG1 among the antigen-specific titers at two weeks after the last vaccination (Figure 3a and Figure S5). However, the IgG2b and IgG3 titers to different vaccine antigens were significantly correlated, except for Prn-specific antibodies (Figure 3a and Figure S5). This suggests that antigen-dependent factors influence the magnitude of IgG1 whereas the mechanisms underlying the magnitude of IgG2b and IgG3 are largely antigen-independent. In other words, a high titer of IgG1 to one of the vaccine antigens did not correlate with a high titer of IgG1 to another vaccine antigen. On the other hand, IgG2b and IgG3 had a stronger correlation of antibody titers to different vaccine antigens. This pattern was reflected in the genetic association mapping as depicted by Venn diagrams (Figure S7). While there were no common genes shared among antigens for IgG1, 570 genes were shared among the four antigens for IgG2b and one gene for IgG3. There was no overlap between the common genes identified for IgG2b and IgG3. These results further suggest that the mechanisms governing the magnitude of IgG titers are antigen dependent for IgG1 but related stronger to factors other than antigens for IgG2b and IgG3. The lack of overlap between subclasses suggests that the genetic factors that influence the production of IgG2b and IgG3 are subclass-specific.

In contrast to the antibody titers, the longevities of IgG1 and IgG2b against the four vaccine antigens were significantly correlated (Figure 3b and Figure S6), suggesting that these are determined by antigen-independent mechanisms. The longevity of IgG3 showed mixed results for their correlation significance (Figure 3b and Figure S6). When using genetic association mapping to validate these findings, all subclasses had genes shared among vaccine antigens with 74 common genes for IgG1, 47 for IgG2b, and 26 for IgG3 (Figure S7). However, there was no overlap across IgG subclasses among these gene sets. These results further support the notion that the mechanisms governing the longevity of IgG are predominantly antigen-independent across IgG subclasses but also suggest that different genes are associated with the longevity for each specific subclass. 

### 3.4. Utilization of the Ratio of IgG1 and IgG3 Titers to Identify Th1 and Th2-Prone Mouse strains

**Figure 4:**
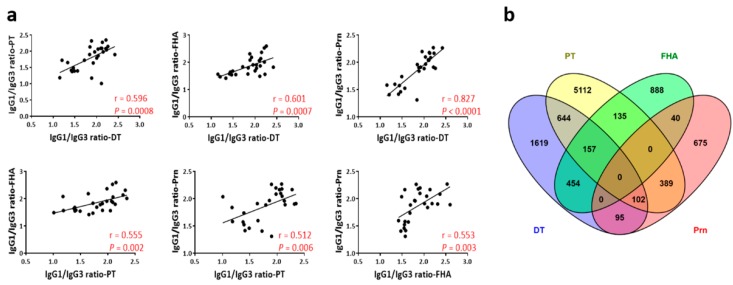
IgG1/IgG3 ratios for different vaccine antigens. (**a**) IgG1 to IgG3 ratios were calculated as log(IgG1/IgG3) for titers measured at wk14 of age. The calculated Pearson correlation coefficients of IgG1/IgG3 ratio (average value of each strain) between the four DTaP antigens were all significant as indicated by the *p*-values. (**b**) Number of genes identified by genome-wide association mapping algorithm for IgG1/IgG3 ratio were plotted by Venn diagram.

**Figure 5:**
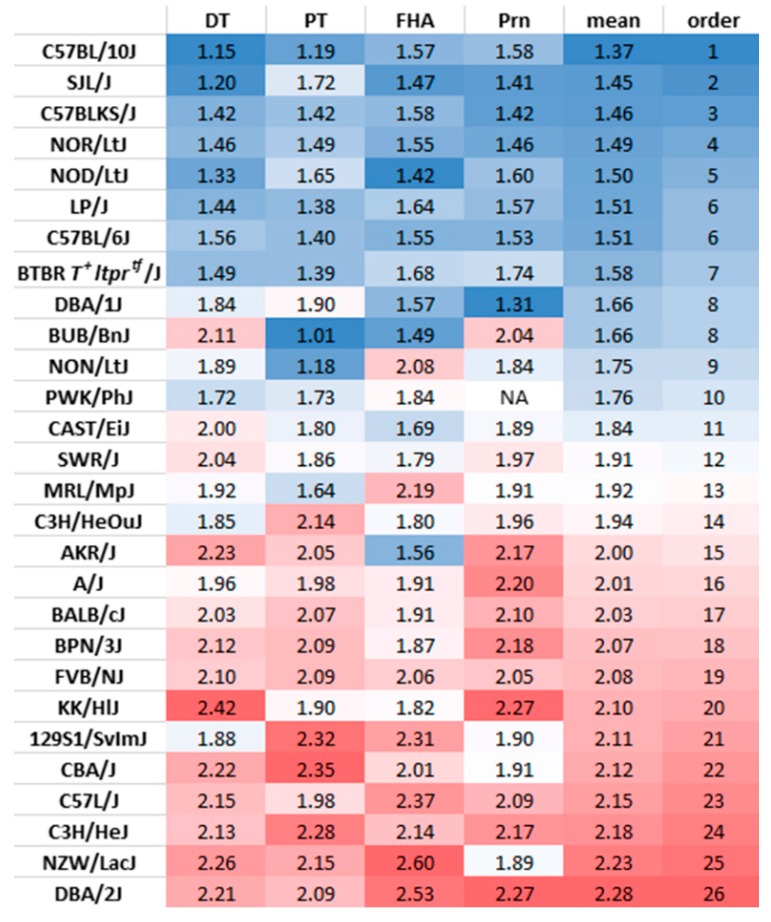
Ranking of mouse strains based on the mean of the IgG1/IgG3 ratios of the four vaccine antigens. A low IgG1/IgG3 ratio indicates Th1-prone and a high ratio indicates Th2-prone. NA: Antibodies induced against Prn in PWK/PhJ mice were below the cutoff and this was eliminated from the analysis.

The ratio of IgG1 and IgG3 antibody titers at wk14 (2 weeks after the third vaccination) was calculated to determine variation among mouse strains in the bias of the immune response to the vaccine towards a Th1 or Th2 response. IgG1 is correlated with Th2 response in the mouse, whereas IgG3 is associated with IFNγ production during a Th1 response [12,15]. We first verified if IgG1/IgG3 ratios could represent Th1 or Th2-biased responses by comparing the ratio between Th1-prone C57BL/6J and Th2-prone BALB/cJ mice. Indeed, the ratio of IgG1/IgG3 was consistently higher, consistent with a Th2 response, in BALB/cJ than C57BL/6J for antibodies measured against all four vaccine antigens at wk14 of age (Figure S8). When the Pearson’s correlation was examined across vaccine antigens, the IgG1/IgG3 ratios consistently showed significant medium to high correlations (Figure 4a), indicating antigen-independent regulatory mechanisms. However, genetic association mapping showed a lack of common genes across the four antigens (Figure 4b). The IgG1/IgG3 ratios averaged for the four vaccine antigens was used to rank the strains from Th1 prone to Th2 prone (Figure 5). C57BL/10J was identified as the most Th1 prone and the DBA/2J as the most Th2 prone among the 28 strains analyzed in this study. C57BL/6J was co-ranked with LP/J as the 6th most Th1-prone mouse strain and the BALB/cJ was ranked as 17th among the 28 inbred strains (Figure 5).

### 3.5. The Effect of TLR4 Signaling on IgG Subclasses

**Figure 6:**
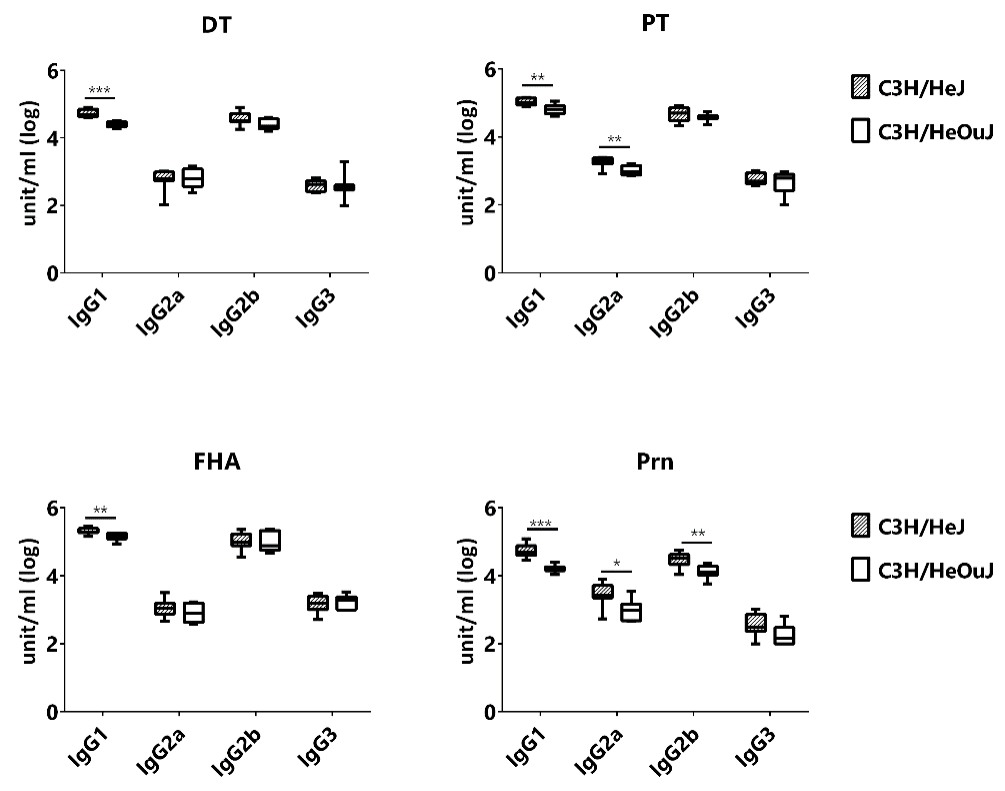
Effect of TLR4 on the magnitude antigen-specific IgG subclass titers in mice immunized with DTaP. Antibody titers at wk14 of age were compared between C3H/HeJ (TLR4-deficient) and C3H/HeOuJ (TRL4-sufficient) mice. * *p* < 0.05; ** *p* < 0.01; *** *p* < 0.005.

The inclusion of both C3H/HeJ (TLR4-deficient) and C3H/HeOuJ (TLR4-sufficient) mice in the cohort allowed us to examine the effect of TLR4 on the production and maintenance of IgG subclasses. Since these two strains share all four IgG subclasses, we measured IgG1, IgG2a, IgG2b and IgG3 antibody titers. Overall, C3H/HeJ produced either similar or higher amount of IgG antibodies. Among the IgG subclasses, the production of IgG1 was consistently higher in C3H/HeJ than that of C3H/HeOuJ for all four antigens (Figure 6). The ratios of DT-specific IgG1/IgG2a and IgG1/IgG3 were both significantly greater for DT in C3H/HeJ mice, suggesting a slight skewing towards a Th2 response, but this was not the case for pertussis antigens (Figure S9). 

## 4. Discussion

In this study, we investigated the antibody response to DTaP vaccination in 28 inbred mouse strains for the magnitude and longevity of IgG subclasses. Twin studies have demonstrated a significant heritable component in the immune response to vaccines [22]. However, these studies only examined total antibody titers and lack data concerning the effect of genetic factors on IgG subclasses and antibody longevity. Our studies reveal variations in antibody magnitude and longevity across mouse strains in a well-controlled environment and the same age, sex, and vaccination regimen, supporting the notion that genetic factors contribute to the heterogeneous vaccine reaction in both antibody production and maintenance including those of different IgG subclasses.

We investigated possible mechanisms underlying the variations in the IgG subclass response to DTaP vaccine antigens by statistical methods, such as Pearson correlation coefficient and GEMMA. Variations in genetic and phenotypic parameters can be used to discover correlations for prediction or generation of mechanistic hypotheses [38]. While the correlations of phenotypes from vaccine responses provide a generalized feature for each phenotype, GEMMA further identified the genetic factors that could underlie the variations of vaccine responses. Our analyses showed that antigen-specific mechanisms drive antibody production of the IgG1 subclass, while variations in IgG2b and IgG3 titers were antigen-independent. This was consistent with the fact that gene association analysis revealed shared genes for IgG2b and IgG3, but not for IgG1. Similar to IgG1, variations in antibody titers for total IgG were also antigen-specific [26] which is not surprising since IgG1 is the dominant subclass produced in response to the DTaP vaccine. The data suggest that antigen-independent factors control the longevity of IgG1, IgG2b, and IgG3 which is consistent with our earlier report for total IgG [26]. It should be noted that different thresholds for significant genetic associations were used in the previous study compared with the current study, and the specific genes cannot be directly compared. In addition, further testing and confirmation of the gene lists is necessary given the relatively low power in genome-wide significance of this approach. 

A possible antigen-independent factor that may influence the longevity of the antibody response is the vaccine adjuvant, which is aluminum hydroxide adjuvant in the DTaP vaccine used in this study. The resurgence of pertussis in spite of excellent vaccination coverage has been attributed to a shorter duration of protection induced by the DTaP vaccine compared with the DTwP vaccine [39]. It has been proposed that a different adjuvant than aluminum hydroxide may induce a longer lasting immune response [40,41]. Very little is known about genetic influences on the response to adjuvants. One study reported differences in the extent of inflammation at the injection site between two inbred strains of rats, suggesting that genetic factors contribute to these differences [42].

The DTaP vaccine used in this study contains protein antigens adjuvanted with aluminum hydroxide which induces a Th2-biased immune response characterized in the mouse by IgG1 antibodies [43,44]. IgG1 was the dominant subclass produced across mouse strains in the current study consistent with other reports [34,35]. The IgG1/IgG2a ratio is a commonly used correlate of Th1 and Th2 responses in mice [43,44]. However, IgG2a is replaced by the allelic variant IgG2c in certain mouse strains [45]. The amino acid sequences of IgG2a and IgG2c differ by 16% and antibodies specific for IgG2a do not or only partially cross-react with IgG2c [45]. It was, therefore, not possible to compare IgG1/IgG2a ratios across the 28 mouse strains in this study, and we chose instead to determine IgG1/IgG3 ratios because the production of IgG3 is dependent on the Th1 cytokine IFNγ similar to IgG2a [46,47]. The IgG1/IgG3 ratio separated C57BL/6J and BALB/cJ mice as expected, suggesting that this measure can be used to rank strains in terms of Th1 versus Th2 propensity. The ranking revealed strains with a stronger Th2 or Th1 bias than BALB/cJ and C57BL/6J mice in response to the aluminum hydroxide adjuvanted DTaP vaccine. The highest IgG1/IgG3 ratio was observed in DBA/2J mice consistent with the strong Th2 response reported previously in T cells from this strain [36]. Further experiments using direct measures of T cell cytokine secretion are necessary to confirm whether the ranking of the mouse strains illustrated in Figure 5 is consistent with Th1 and Th2 responses. Immunization of children with a DTaP vaccine induces pertussis antigen-specific IgG predominantly comprised of IgG1 followed by IgG4 with a variable proportion of IgG2 and a negligible amount of IgG3 [48,49,50]. The induction of IgG4 antibodies was attributed to the aluminum adjuvant as this isotype is associated with Th2 responses in people [51,52]. There was substantial variability in IgG4 titers among children, which may reflect genetic variation in the response to the DTaP vaccine. 

The antibody concentration in serum is affected by the rates of synthesis and catabolism of immunoglobulin molecules [53,54]. IgG is different from other isotypes as it has a longer half-life. The IgG protection receptors originally proposed by Brambell in 1964 [55] were later identified as the neonatal Fc receptors (FcRn) [56,57,58]. The FcRn is expressed by endothelial cells and antigen presenting cells and maintains IgG homeostasis by binding and releasing IgG back into the circulation in a pH-dependent manner [59]. The FcRn appears to have a similar high affinity for all IgG subclasses and there is no evidence for differences in FcRn among mouse strains [60,61]. The variation in antibody longevity among mouse strains can, therefore, most likely be attributed to differences in the rates of antibody synthesis rather than catabolism of IgG. 

We reported previously that TLR4-deficient C3H/HeJ mice produced higher IgG titers against the four vaccine antigens than the TLR4-sufficient C3H/HeOuJ mice [26]. Similar results were obtained for IgG1 in the current study when comparing between C3H/HeJ and C3H/HeOuJ. The lack of TLR4 caused a modest shift towards Th2 bias based on the increased IgG1/IgG2a and IgG1/IgG3 ratios, which was only significant for DT. The immunostimulatory effect of aluminum adjuvants is independent of MyD88 and TRIF, the adaptor molecules associated with Toll-like receptors, including TLR4 [62]. The DTaP vaccine is devoid of the classical TLR4 agonist LPS, but both intact and inactivated PT as well as the PTB subunit can activate TLR4 signaling pathways [63,64,65]. The modest effect of TLR4-deficiency and overall Th2-biased response induced by the DTaP vaccine suggests that the PT in the vaccine formulation has weak biological activity. 

## 5. Conclusions

Genetically diverse inbred mouse strains differed markedly in the production and longevity of the IgG subclasses induced by vaccination with DTaP. The production of IgG1 was antigen-dependent. However, the decline of antibody titers for IgG1, IgG2b, and IgG3 was largely antigen-independent, suggesting that common factors in the vaccine formulation instead of antigen-specific factors determine the longevity of the antibody response. We further show that IgG1/IgG3 ratios could be a useful measure to represent Th1/Th2 vaccine responses across mouse strains.

## Supplementary Materials

The following are available online at https://www.mdpi.com/2076-393X/7/4/124/s1, Figure S1: Longevity of IgG1, IgG2b and IgG3 to DTaP vaccine antigens in inbred mouse strains, Figure S2: IgG1, IgG2b and IgG3 titers at 24 weeks of age, 12 weeks after the last vaccination, Figure S3: Correlations between the magnitude of IgG1 and IgG2b (a), IgG1 and IgG3 (b), IgG2b and IgG3 (c) titers against the same vaccine antigen, Figure S4: Correlations between the longevity of IgG1 and IgG2b (a), IgG1 and IgG3 (b), IgG2b and IgG3 (c) titers against the same vaccine antigen, Figure S5: Correlations between the magnitude of IgG1 (a), IgG2b (b) and IgG3 (c) titers against different vaccine antigens, Figure S6: Correlations between the longevity of IgG1, IgG2b and IgG3 titers against different vaccine antigens, Figure S7: Venn diagrams for common genes associated with antibody titers and longevity, Figure S8: IgG1/IgG3 ratio against different vaccine antigens in BALB/cJ and C57BL/6J mice, Figure S9: Effect of TLR4-deficiency on the IgG1/IgG2a and IgG1/IgG3 ratios in mice immunized with DTaP. Excel files: Gene lists for week 14 titers IgG1, week 14 titers IgG2b, week 14 titers IgG3, longevity IgG1, longevity IgG2b, longevity IgG3, IgG1/IgG3 ratio.
