# Supplementary material for: Genetic Variation in the Magnitude and Longevity of the IgG Subclass Response to a Diphtheria-Tetanus-Acellular Pertussis (DTaP) Vaccine in Mice
Source: Vaccines (Basel). 2019 Sep 20;7(4):124. doi: 10.3390/vaccines7040124 (PMC6963843; doi:10.3390/vaccines7040124)
Supplement: Supplementary file 1 [file vaccines-07-00124-s001.zip › Supplemental Data/Mosley Supplementary Figures Sep 3 2019.pdf]

Supplementary Figures

Genetic variation in the magnitude and longevity of the IgG subclass response to a Diphtheria-Tetanus-acellular Pertussis (DTaP) vaccine in mice

Yung-Yi C. Mosley, Josiah E. Radder, Harm HogenEsch

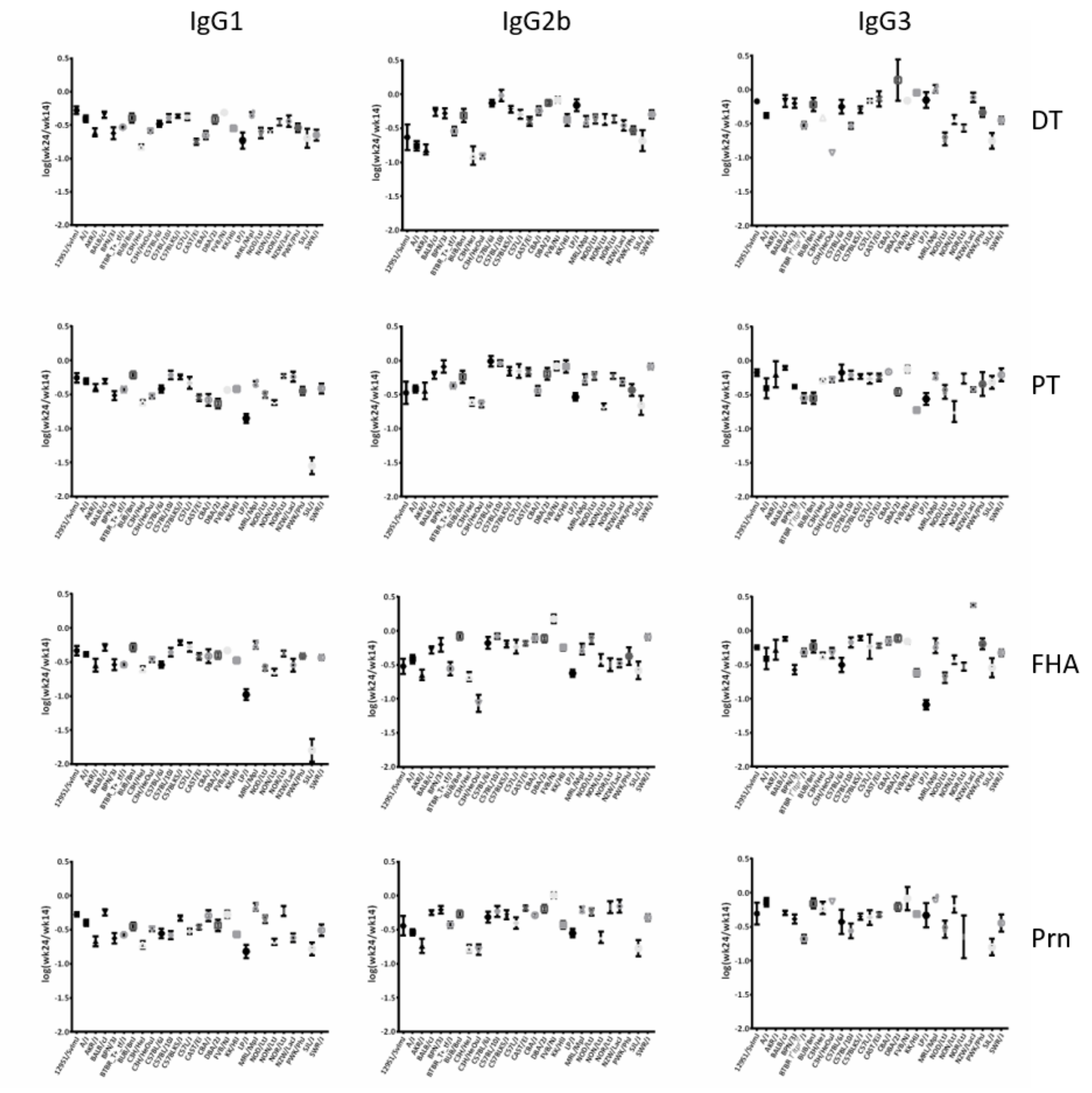

Fig. S1   Longevity of IgG1, IgG2b and IgG3 to DTaP vaccine antigens in inbred mouse strains. The antibody longevity was defined as the log_{10} ratio of IgG subtype titers at week 24 versus week 14. There was a significant difference among mouse strains (*P* < 0.0001 by ANOVA) for antibody longevity against diphtheria toxoid (DT), pertussis toxin (PT), filamentous hemagglutinin (FHA), and pertactin (Prn) for each IgG subtype. Mouse strains are presented in alphabetical order. Data were included for all mouse strains except DBA/1J.

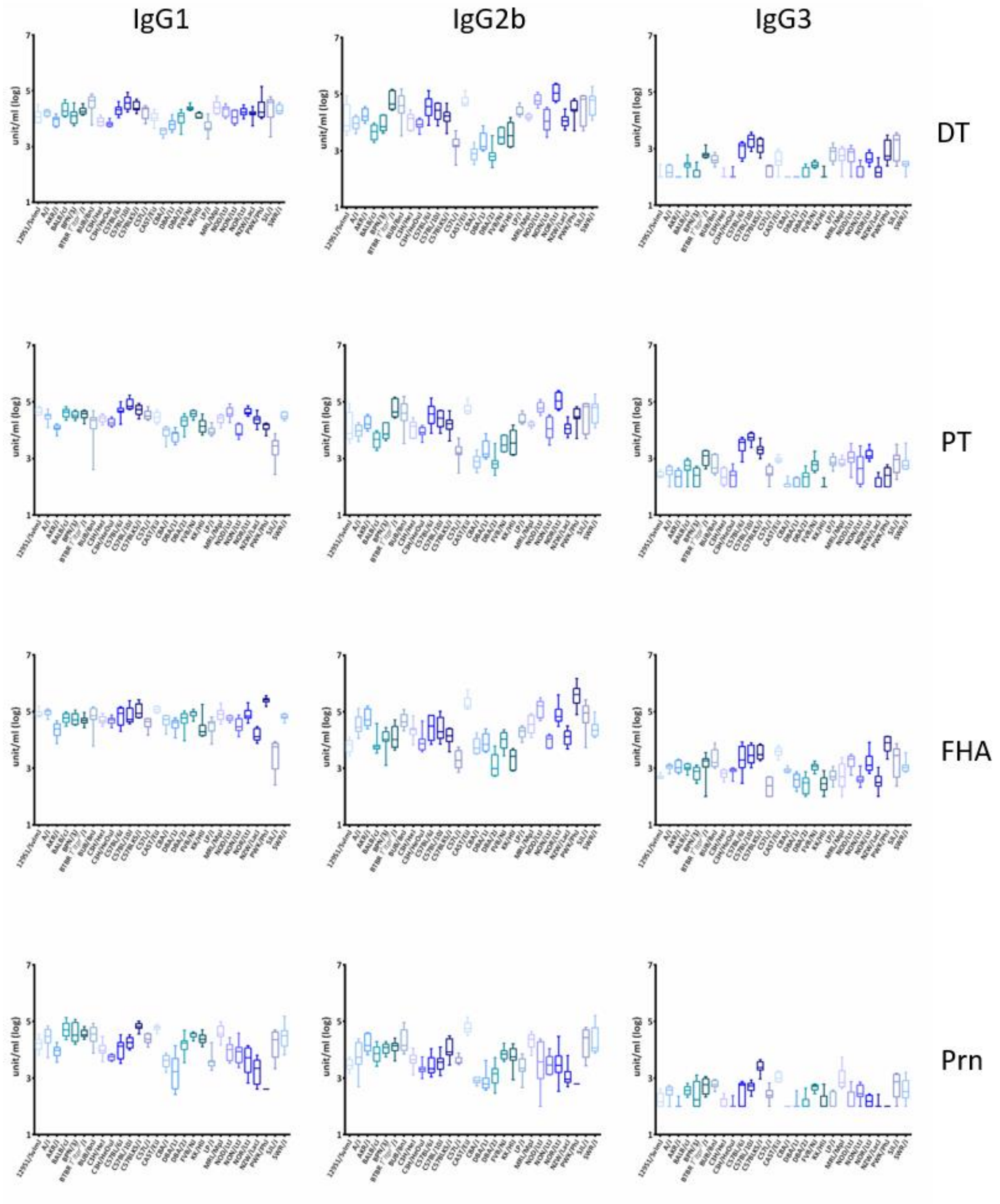

Fig. S2 IgG1, IgG2b and IgG3 titers at 24 weeks of age, 12 weeks after the last vaccination.

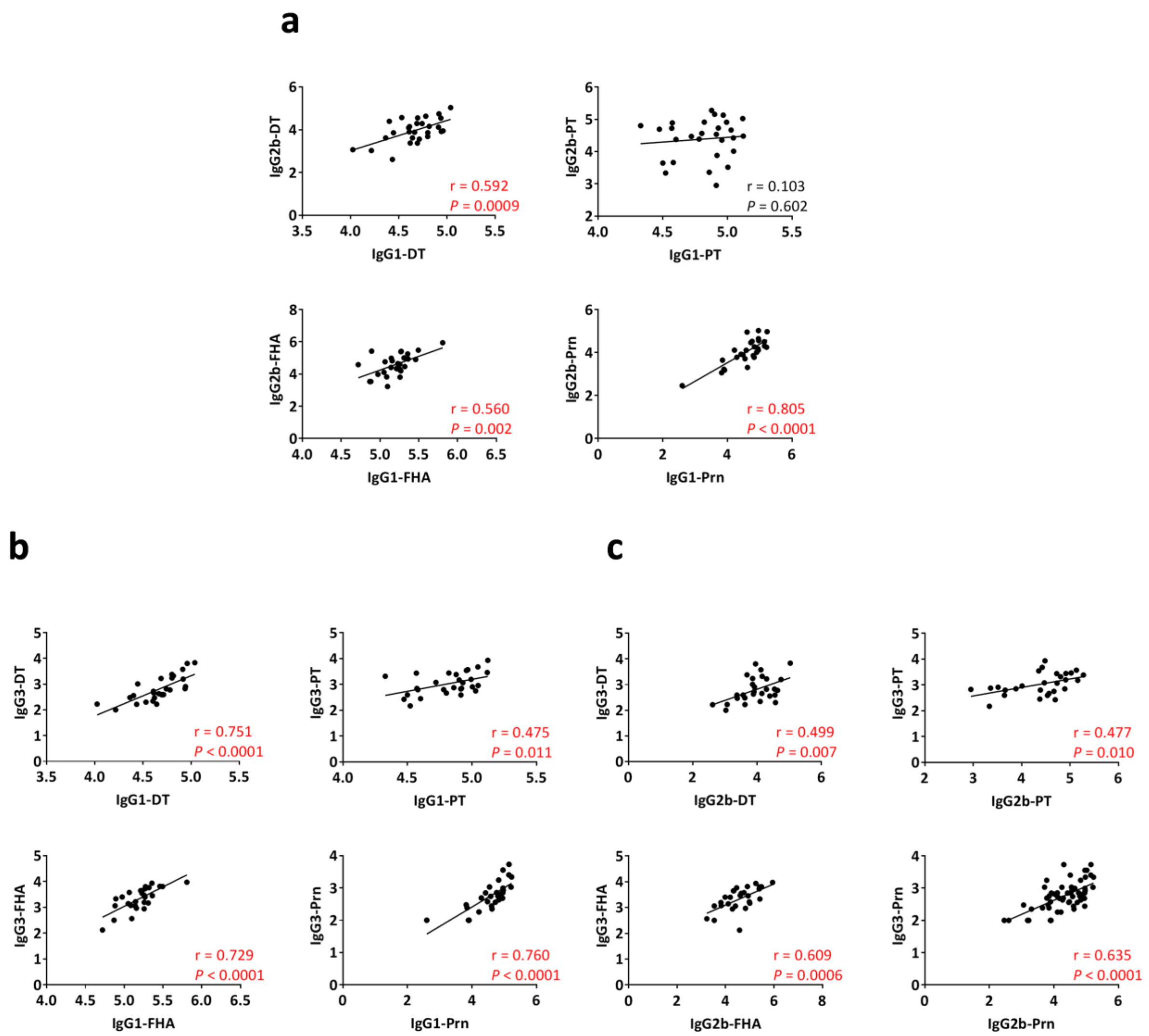

Fig. S3. Correlations between the magnitude of IgG1 and IgG2b (a), IgG1 and IgG3 (b), IgG2b and IgG3 (c) titers against the same vaccine antigen. Mean value of the magnitude for each strain was used to calculate the Pearson's correlation coefficient (r). Significant values (*p* <0.05) are indicated in red.

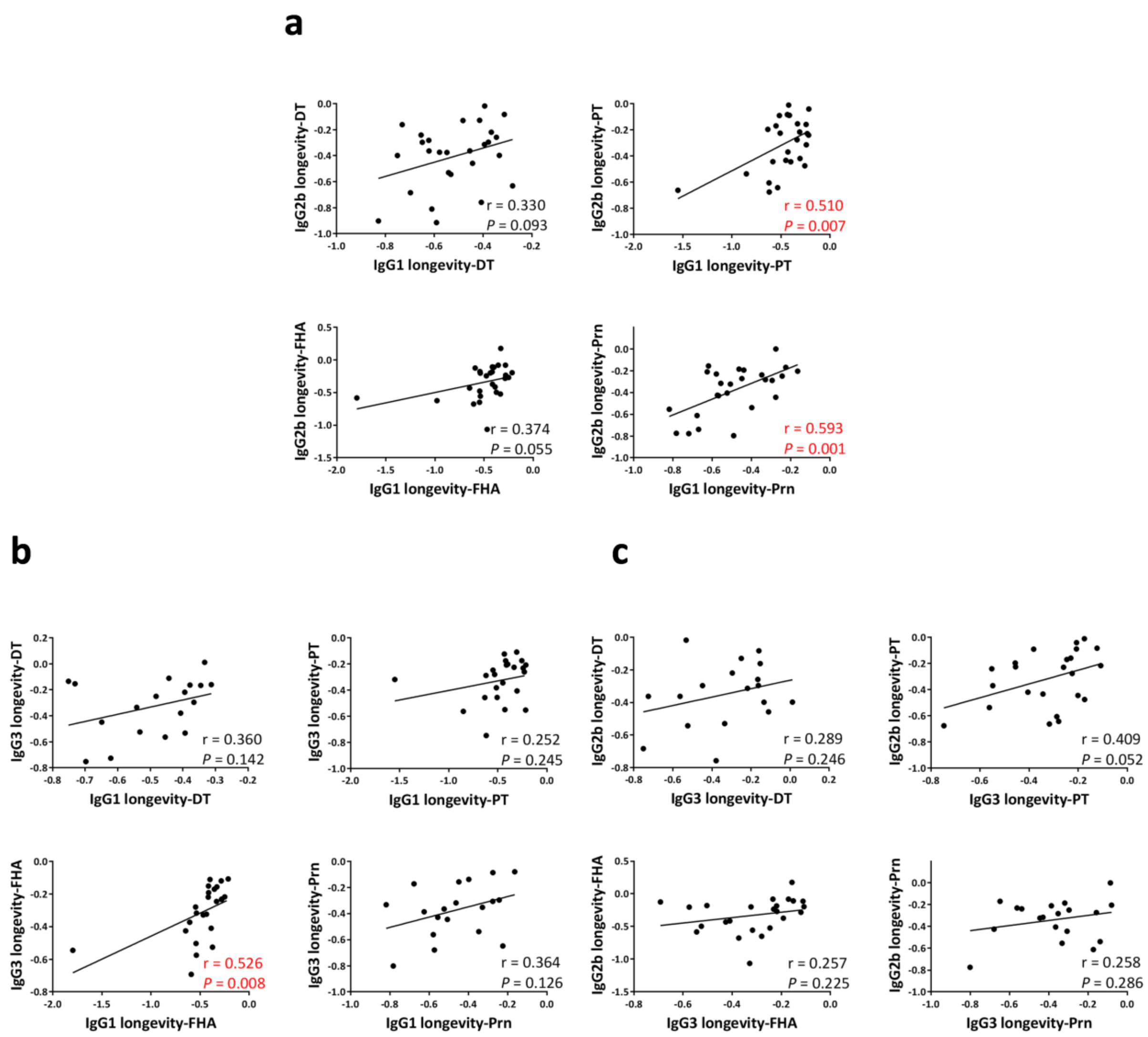

Fig. S4 Correlations between the longevity of IgG1 and IgG2b (a), IgG1 and IgG3 (b), IgG2b and IgG3 (c) titers against the same vaccine antigen. Mean value of the longevity of each strain was used to calculate for Pearson's correlation coefficient (r). Significant values ($p < 0.05$) are indicated in red.

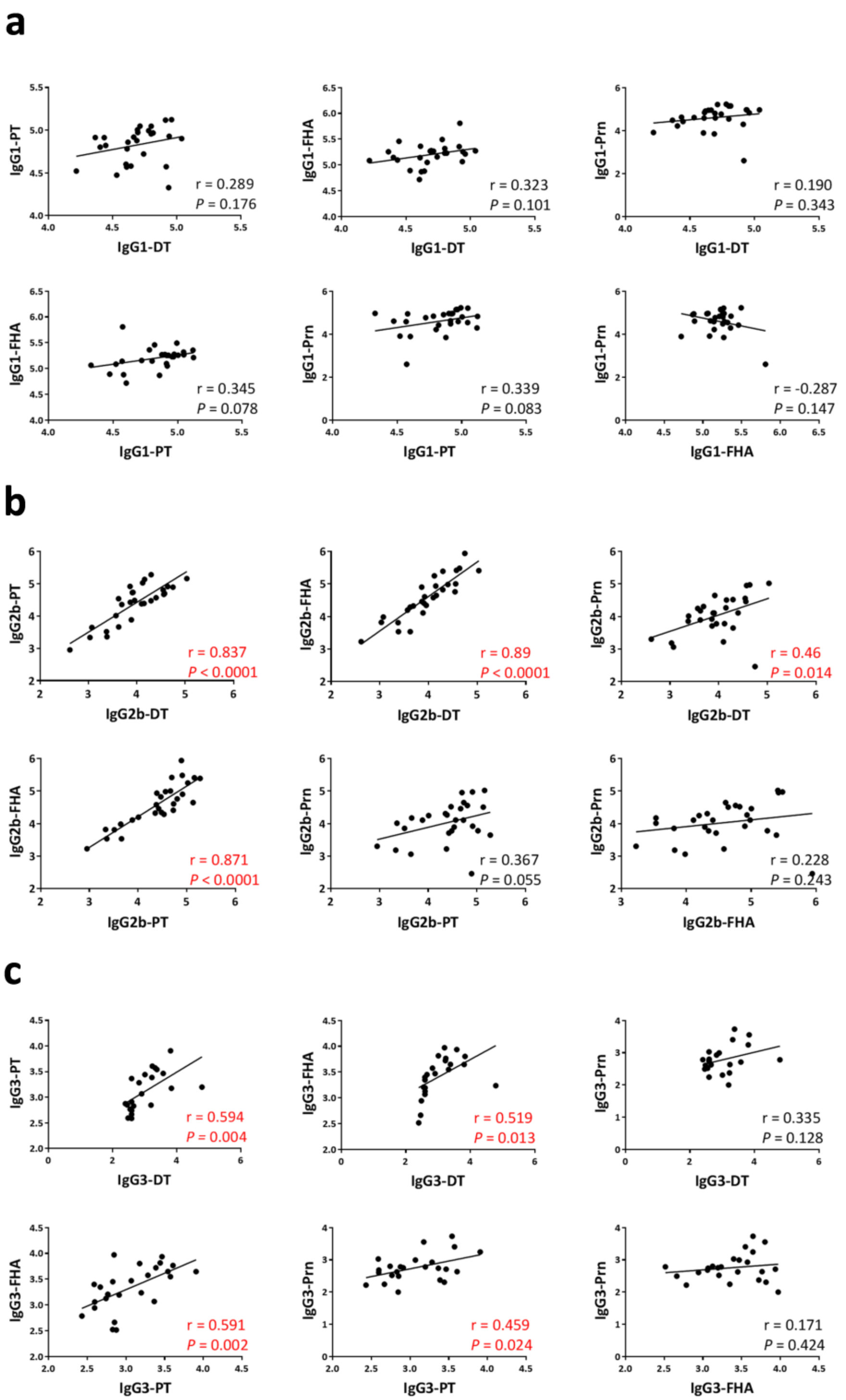

Fig. S5 Correlations between the magnitude of IgG1 (a), IgG2b (b) and IgG3 (c) titers against different vaccine antigens. Significant correlations are in red.

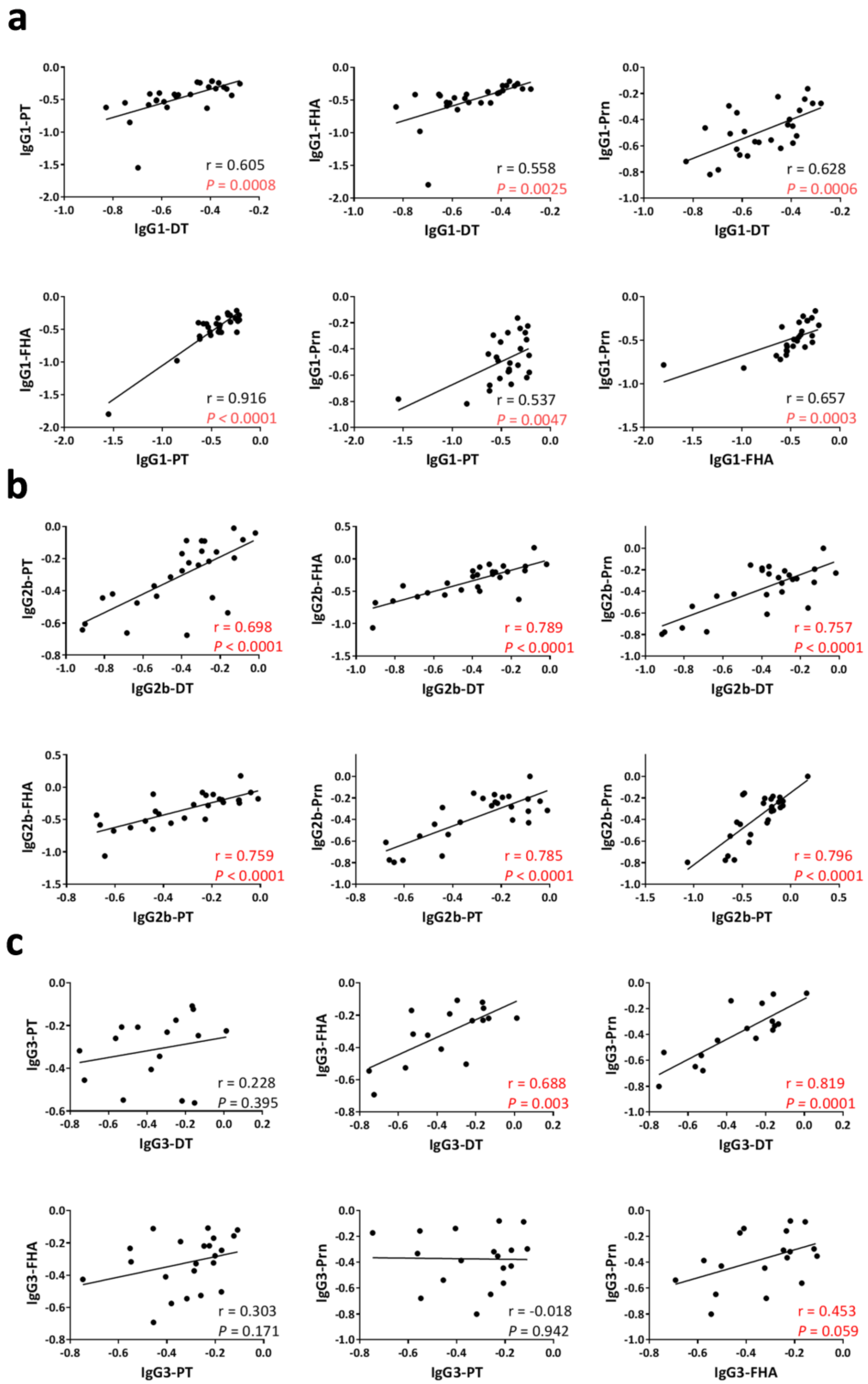

Fig. S6 Correlations between the longevity of IgG1, IgG2b and IgG3 titers against different vaccine antigens. Significant correlations are in red.

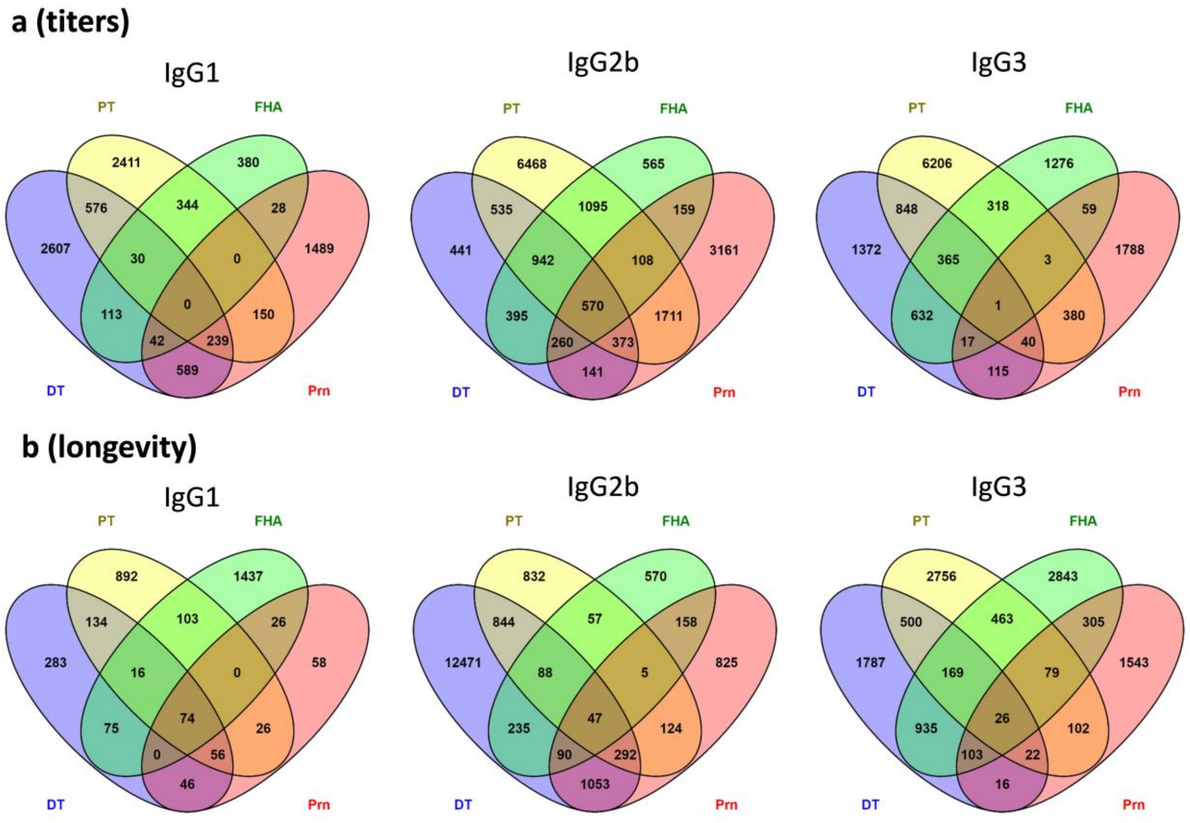

Fig. S7 Venn diagrams for common genes associated with antibody titers and longevity. Genes identified by the association mapping algorithm for each IgG subclass were plotted by Venn diagram for antibody titers (a) and longevity (b). There are common genes shared by antibody against different vaccine antigens for antibody magnitude of IgG2b and IgG3 and for antibody longevity of IgG1, IgG2b and IgG3.

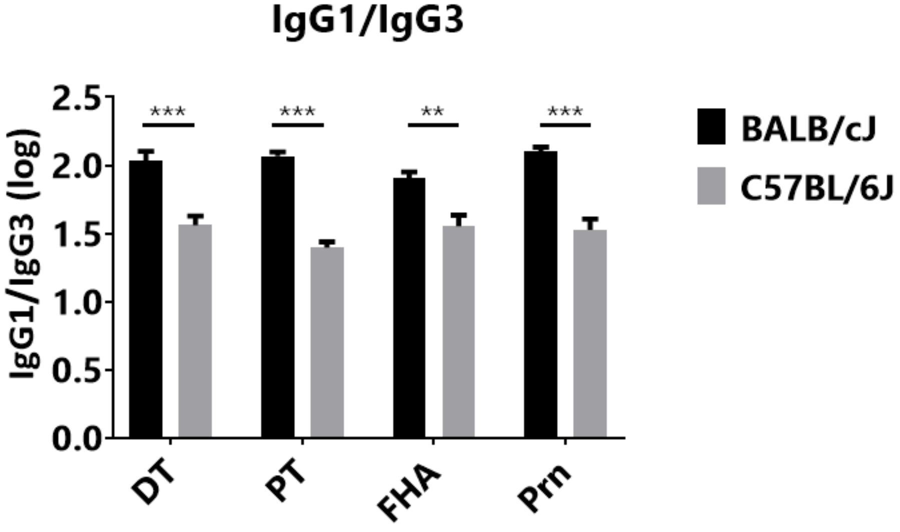

Fig. S8. IgG1/IgG3 ratio against different vaccine antigens in BALB/cJ and C57BL/6J mice.
The IgG1 to IgG3 ratio was calculated as log10 (IgG1/IgG3) for titers measured at wk14 of age. The IgG1/IgG3ratio of BALB/cJ was significantly higher than that of C57BL/6J strain for all four antigens. ** $p < 0.01$; *** $p < 0.005$.

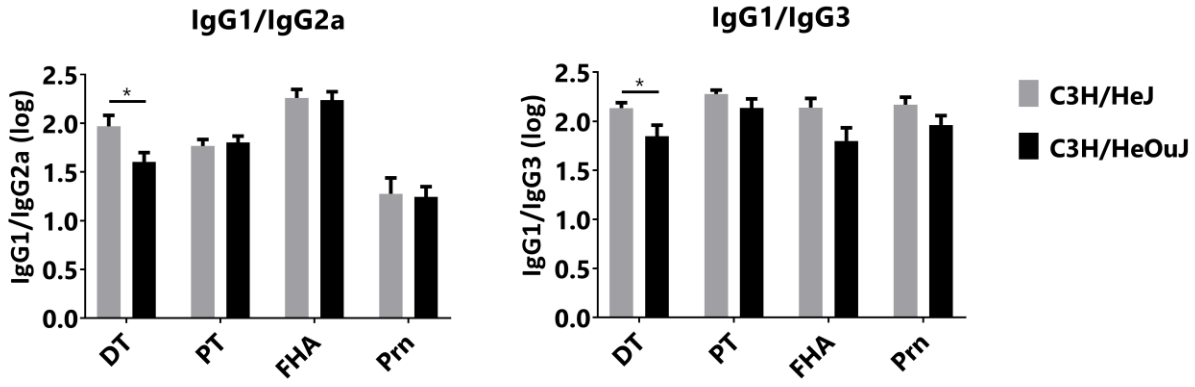

Fig. S9 Effect of TLR4-deficiency on the IgG1/IgG2a and IgG1/IgG3 ratios in mice immunized with DTaP. The IgG1/IgG2a and IgG1/IgG3 ratios were calculated as $\log_{10}$ (IgG1/IgG3) for titers measured at wk14 of age. * $p < 0.05$
